# Recommendations for and compliance with social restrictions during implementation of school closures in the early phase of the influenza A (H1N1) 2009 outbreak in Melbourne, Australia

**DOI:** 10.1186/1471-2334-11-257

**Published:** 2011-09-30

**Authors:** Jodie McVernon, Kate Mason, Sylvia Petrony, Paula Nathan, Anthony D LaMontagne, Rebecca Bentley, James Fielding, David M Studdert, Anne Kavanagh

**Affiliations:** 1 Vaccine & Immunisation Research Group, Murdoch Children's Research Institute and Melbourne School of Population Health, University of Melbourne, Australia; 2 Centre for Women's Health, Gender and Society, Melbourne School of Population Health, University of Melbourne, Australia; 3 McCaughey Centre, Melbourne School of Population Health, University of Melbourne, Australia; 4 Victorian Infectious Diseases Reference Laboratory, North Melbourne, Australia; 5 Victorian Government Department of Health, Melbourne, Australia; 6 Centre for Health Policy, Programs and Economics, Melbourne School of Population Health, University of Melbourne, Australia; 7 National Centre for Epidemiology & Population Health, The Australian National University, Canberra, Australia; 8 Melbourne Law School, University of Melbourne, Australia

## Abstract

**Background:**

Localized reactive school and classroom closures were implemented as part of a suite of pandemic containment measures during the initial response to influenza A (H1N1) 2009 in Melbourne, Australia. Infected individuals, and those who had been in close contact with a case, were asked to stay in voluntary home quarantine and refrain from contact with visitors for seven days from the date of symptom onset or exposure to an infected person. Oseltamivir (Tamiflu^®^) was available for treatment or prophylaxis.

**Methods:**

We surveyed affected families through schools involved in the closures. Analyses of responses were descriptive. We characterized recommendations made to case and contact households and quantified adherence to guidelines and antiviral therapy.

**Results:**

Of the 314 respondent households, 51 contained a confirmed case. The prescribed quarantine period ranged from 1-14 days, reflecting logistic difficulties in reactive implementation relative to the stated guidelines. Household-level compliance with the requirement to stay at home was high (84.5%, 95% CI 79.3,88.5) and contact with children outside the immediate family infrequent.

**Conclusions:**

Levels of compliance with recommendations in our sample were high compared with other studies, likely due to heightened public awareness of a newly introduced virus of uncertain severity. The variability of reported recommendations highlighted the difficulties inherent in implementing a targeted reactive strategy, such as that employed in Melbourne, on a large scale during a public health emergency. This study emphasizes the need to understand how public health measures are implemented when seeking to evaluate their effectiveness.

## Background

The World Health Organization declared the first influenza pandemic of the 21^st ^Century in June 2009, following global spread of a novel swine-origin reassortant strain of influenza A (H1N1) (pH1N1) [1]. In Australia, as in many countries, initial reports were dominated by outbreaks in schools, with evidence of high rates of transmission between children [2]. Anticipating the special risks posed in the school environment, the Australian Health Management Plan for Pandemic Influenza 2008 (AHMPPI) [3] had recommended school and classroom closures as part of a suite of 'social distancing' measures aimed at limiting early spread of an imported pandemic virus. Other interventions during the initial 'Contain' phase of the pandemic response included voluntary home quarantine of cases and their close contacts, and liberal distribution of antiviral agents for treatment and prophylaxis of infection [3].

Although school closure has been widely used in the response to past pandemics [4], there is little quantitative evidence of its likely effectiveness to inform optimal implementation [5]. This absence of data is particularly troublesome given the estimated societal costs of school and workplace closures in the context of a pandemic response as predicted by macroeconomic models [6,7]. Mathematical models have been used to estimate the impact of school closure on epidemic dynamics, with disparate conclusions. These variations arise because of differing assumptions regarding relative age-specific attack rates [8], social mixing patterns prior to [9] and during [5] the period of school closure and the timing and extent of interventions [10,11]. Within these model frameworks, full compliance with voluntary home quarantine recommendations is often assumed, perhaps erroneously given perceived inconvenience [12]. Even where models find simulated school closures to be effective at reducing disease, their associated societal costs generally exceed savings to the health care system resulting from case prevention [13].

Localized reactive school and classroom closures were employed during the Contain phase of the response to pH1N1 in metropolitan Melbourne, Victoria, Australia, between 22^nd ^May and 3^rd ^June 2009 [14]. In Victoria, Department of Health guidelines recommended that schools with multiple confirmed cases in different classes should be closed for seven days from the date that the last confirmed case attended school; schools with confirmed cases in one class were instructed to close only that class. Quarantined individuals were asked to stay at home and refrain from contact with visitors for seven days from the date of symptom onset or exposure to an infected person - in the first week of measures the recommended period may have been as long as fourteen days in some cases (J Fielding, personal communication).

This questionnaire-based study aimed to characterize the implementation of this intervention across all schools that enacted closures in the Melbourne metropolitan area, representing a population of 4.1 million residents. We also sought to quantify adherence to behavioural and pharmaceutical recommendations, and define household characteristics associated with differences in compliance.

## Methods

### Study population

In the state of Victoria, the three main education providers are the State Government (1613 schools), Catholic Education (484 schools) and the Independent schools sector (692 schools)(http://www.australianschoolsdirectory.com.au/educationinformation.php?region=28)We obtained from the Victorian government departments of Health and Education and the Catholic Education Office lists of government and Catholic schools in which closures were implemented from the 22^nd ^May to 3^rd ^June 2009. From these lists, we identified a total of 82 potentially affected schools. Discussions with the principals at these schools regarding the pandemic response confirmed that only 39 had effected closures, and 33 of these agreed to participate - 6 schools did not respond to our enquiry (85% school participation rate). The reasons for differential reporting of school closure status by government agencies and principals were not clear.

On our behalf, staff at participating schools forwarded study information to the parents of 1,181 students in the closed classes or teaching groups who had been advised to go into voluntary home quarantine. An initial letter and two reminder letters were sent to each identified family during November 2009. The second reminder included a movie voucher valued at $AU10.30 to boost participation and thank families for their involvement. Participating schools received $AU20 towards the purchase of educational resources for each completed questionnaire.

In Australia, each school is characterized according to a national 'Index of Community Socio-Educational Advantage' (ICSEA), a measure that incorporates Australian Bureau of Statistics data (such as parental incomes, education and employment), Aboriginal enrolment data and community remoteness - all factors known to predict educational outcomes (http://www.myschool.edu.au). Students are allocated to quartiles of advantage relative to the national average. If a school has a disproportionate number of students in the lowest quartile, it is likely to be serving a very disadvantaged community. We looked for a relationship between the response rate at school level and the difference between the proportion of students in the lowest quartile and the national average of 25%, using univariate linear regression.

The study was approved by the University of Melbourne's Health Sciences Human Ethics Sub-Committee (0932293). The Department of Education and Early Childhood Development and the Catholic Education Office granted permission for us to approach schools to conduct the survey.

### Survey

Participating parents completed an anonymous online or telephone questionnaire, which elicited a range of information, including: the compliance of all family members with behavioural recommendations and pharmaceutical interventions during the quarantine period, and factors that may have influenced compliance such as parental leave entitlements and attitudes to the intervention. This study focuses on quantitative measures of compliance. A copy of the questionnaire is available from the authors on request.

### Measures of compliance

Compliance with home quarantine was calculated as a proportion, representing the number of days spent at home, divided by the number of recommended days of quarantine (i.e. voluntary self-quarantine beyond this period was not assessed). This measure was derived for each individual, along with the proportion of individuals who stayed at home for all of their recommended quarantine days. Compliance was further assessed at household level, representing the number of recommended quarantine days on which all family members who were asked to go into quarantine complied with recommendations. Respondents were asked to identify any trips made outside the home by quarantined individuals, whether the trips were to open or enclosed public spaces, and whether other persons were present.

Compliance with social mixing recommendations was assessed by asking whether adults or children who were not members of the households made incursions to the home environment lasting more than 15 minutes during the quarantine period. For each day nominated as being spent outside the home, the questionnaire elicited information on any mixing with children who were not family members. In any care location, participants were also asked to state whether primary child carers normally lived with the child or were from another household.

For every family member who was prescribed oseltamivir (Tamiflu^®^), respondents were asked whether all, half or more, less than half or none of the course was completed. Reasons for less than full completion were elicited.

### Statistical analysis

Statistical analyses were performed using Stata 11.0. Analyses were performed at the level of either household or individual, depending on the outcome measure. To adjust our estimates of compliance for the clustering of responding households within schools and individuals within households, we used logistic regression modelling and post estimation commands, and reported the estimates as percentages with 95% confidence intervals (95% CIs). P-values are reported for comparisons between groups. Multilevel regression was used to investigate the extent to which the variance in household compliance was attributable to school-level versus household-level differences. Individual-level compliance estimates were adjusted only for clustering of individuals within households, as the clustering of compliance at the school level was estimated to be of minimal impact.

## Results

### Study population

The population of schools surveyed derived from relatively disadvantaged areas, with 16 schools reporting a larger proportion of students in the bottom quartile of advantage according to ICSEA scores than the national average (Median difference: 4, range -25, 39). Median school level response rates were 19.9% (Range: 4%, 46%). Response rates were square root transformed to approximate a normal distribution, and linear regression performed to assess the relationship between this score and the excess (or under-representation) of students in the least advantaged quartile. The two were significantly related (Coefficient (95%CI): -0.04 (-0.06, -0.01); p = 0.002), reflecting lower response rates from less advantaged schools.

**Figure 1:**
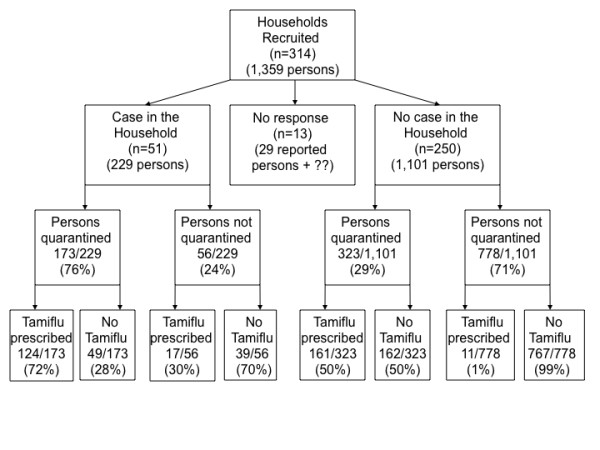
**Quarantine and prophylaxis recommendations, by case status of the household**.

We received 314 responses from 1,181 (27%) eligible households approached by the 33 participating schools. Of these, 301 primary respondents (96%) provided information regarding the presence or absence of a medically diagnosed case in the household. Reporting households ranged in size from 2 to 9 members (median 4, interquartile range 4 to 5) and contained a total of 1,330 persons (Figure 1). The total number of household members in families (n = 13) not reporting case status could not be determined due to missing data. Fifty-one families reported at least one pH1N1-infected individual. Seven of these families reported a secondary case and four reported two secondary cases, for an average secondary household attack rate of 6%. Only one of the 51 primary cases was older than 18 years.

### Quarantine recommendations

**Figure 2:**
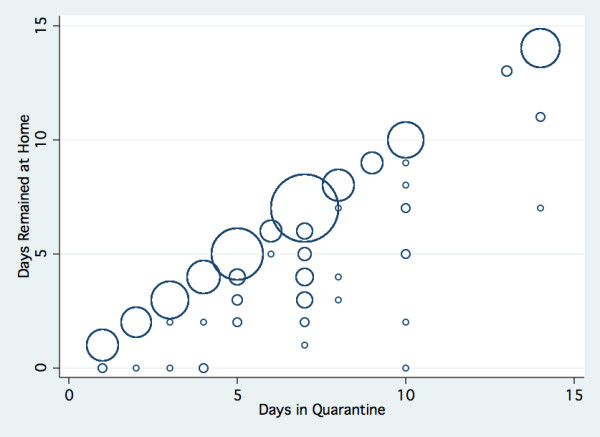
**Days spent at home relative to the recommended duration of quarantine (size of circles reflects the frequency of reported observations)**.

Four hundred and ninety-six individuals were asked to stay in voluntary home quarantine in association with the school and classroom closures. Quarantine was more likely to be recommended for household members if a child had a confirmed case of influenza. The recommended quarantine periods varied, ranging from 1-14 days (median 7 days, IQR 5-8 days) (Figure 2).

### Compliance with requirements to stay at home

Individual compliance with the recommendation to stay at home was high, with respondents reporting that individuals stayed at home for more than 94% of the days they were advised to be in quarantine (95% CI 92.8, 95.9). This figure was not associated with the length of quarantine (Figure 2) and did not fluctuate over the course of the quarantine period (data not shown). Of the 3,232 quarantined days, respondents reported that they and their family members spent most of their time outside the home during only 177 days. Of these days, 47 were spent in the homes of friends, 44 at school, 18 in the workplace and 68 at 'Other' unspecified locations. The proportion of individuals who remained at home during all days of their prescribed quarantine period was 88% - this lower figure was attributable to the variable length of the recommended quarantine period for any given individual, as shown in Figure 2.

When compliance was considered at household level, 250 households (84.5%; 95% CI: 79.3%, 88.5%) reported perfect compliance by all family members with quarantine recommendations throughout its duration, regardless of whether there was a case in the household (82.0% compliant) or not (85.0% compliant) (p = 0.57).

We estimated that only one per cent of the variation in this compliance outcome was explained by differences at the school level (level 2 variance), while 99% of variation was due to differences between households (level 1 variance).

### Compliance with restrictions on outings

During the quarantine period, 25 reporting households (8.4%; 95% CI: 0.05%, 12.9%) stated that at least one quarantined family member left the home to visit "an outdoor public space with lots of other people around (e.g. playground or market)". A further 36 respondents (12.0%; 95% CI: 0.08%, 17.0%) reported an excursion to an enclosed public space, other than for medical attendance. There was no significant difference in such incidents between families with or without a resident influenza case (data not shown).

### Compliance with requirements to avoid social mixing

The main purpose of school closure was to restrict contact between children that may facilitate the spread of infection. Forty-three households reported that a child spent at least one day outside the family home, and mixing with other children occurred on almost half of these occasions (48.8%; 95% CI: 35.7%, 62.1%), whether or not there was a case in the family (p = 0.5). Contact with children who were not immediate family members was far less likely during days spent at home. No child visited a study household in which another child was ill, compared with reported child visitors in 15.9% of 226 homes without a case (p < 0.001). Adult visitors were somewhat more common (31.1%; 95% CI: 25.5%, 37.3%), and again occurred more frequently in households without (33.5%) than with (19.6%) an influenza-infected individual (p = 0.04).

Compared to children in households that complied with recommendations to stay at home, children in households that did not comply with the recommendations were more likely to have been cared for during the quarantine period by an adult from outside the home (28.3% compared with 4.0% for compliant households; p < 0.001), thus also contravening the quarantine recommendation not to mix with adults from outside the household. This distinction was especially marked for households in which there was a confirmed case of influenza, where the difference was 44.4% of children receiving outside care in non-compliant households compared with 2.4% of those that were compliant.

### Compliance with antiviral medications

Oseltamivir was prescribed for 313 individuals, more often if there was a case in the household and/or for quarantined persons (Figure 1). Compliance with the medication was high, with 75% of respondents stating that the full drug course was completed (95% CI: 68.2, 80.6%). Only 7.1% refused it altogether, 9.9% took up to half, and 5.1% more than half (2.9% were unsure). The presence of a case in the household did not affect adherence to the prophylaxis or treatment regimen, nor did the age of the individual prescribed the medication. Reasons for non-completion of the course did, however, vary by age (data not shown). Where non-compliance was reported, the primary household respondent attributed this to belief that the drug was unnecessary (n = 42), particularly for individuals older than 18 years (p = 0.02). Some children refused to take the medication for unstated reasons (n = 10), but side effects, experienced (n = 12) or anticipated (n = 8), were infrequently reported.

## Discussion

Despite variable recommendations for the containment of pH1N1 in Victoria (Australia), our findings suggest that compliance with both behavioural and pharmaceutical recommendations was high, particularly in case households. These closures occurred during a well-defined and relatively constricted time frame, at the very beginning of the pandemic strain's emergence in Australia, where Victoria was the first state to report person-to-person transmission. As Australia was one of the first countries to experience pH1N1 outbreaks during the Southern Hemisphere winter, local public health officials were uncertain of the likely severity of disease and acted according to the 'worst case scenario' recommendations of the AHMPPI 2008 during the initial Contain phase. Considerable media attention was focussed on school-based spread of infection and the associated public health response. Our findings may therefore be indicative of a 'best case' estimate of the public's compliance during a moderate to severe influenza pandemic.

Issues arising in the conduct of our survey highlighted the considerable logistic challenges involved in implementing this complex policy on a large scale. In seeking to quantify implementation of school closure measures in Melbourne during the 2009 pH1N1 response, it was first apparent that government records of the intervention did not accord with the level of stated school involvement. Reasons for this discrepancy were unclear, but based on discussions with principals, did not represent school refusal to comply with directives. An alternative explanation might relate to the practical challenges involved in centralized administration of a localized reactive public health intervention, applied across many sites. The highly variable quarantine duration recommended to families provides further support for this hypothesis.

Inevitable delays to response arising from the multiple steps to initiation of closure including: case diagnosis, public health reporting, contact identification and information dissemination were reflected in frequent reports of quarantine periods less than seven days. A quarantine duration of three days or less may not reliably exclude development of infection, given some variation in the length of the presymptomatic infectious period [15], particularly in children [16]. Moreover, as the period of isolation was to extend for *a total of 7 days following last contact with an infected individual*, it must be assumed that those contained for a shorter period had already spent several days post-exposure mixing freely in the community, during the time at which they were most likely to be infectious.

### Strengths and limitations of the study

This is the first study to evaluate implementation of school closure on such a large scale, with our 33 schools representing an intervention conducted across the whole of metropolitan Melbourne. The low response rate from invited participants in this study is consistent with that observed in similar surveys [17-19], but does introduce potential for ascertainment bias. In particular, we received a disproportionately low level of responses from less advantaged schools, limiting our ability to represent the whole population experience and possibly inflating estimates of compliance. Also of note, the proportion of households that contained a confirmed case (20%) was considerably higher than that in a recently published West Australian (WA) study of school closures (5%) [20]. This may suggest that not only more affluent, but also more concerned and/or compliant parents were more likely to take part in our study. Study materials were not available in languages other than English, which may also have excluded vulnerable subgroups in the population sample. Unfortunately, as invitations to participate were distributed through schools due to privacy constraints, we are not able to characterize non-respondent households in more detail. Further, conduct of the survey several months after closures took place may have reduced motivation to participate, and introduced the possibility of recall bias.

### Findings in relation to other studies of quarantine compliance

Why was compliance with quarantine recommendations so high in our sample? The study of school closures in WA, implemented later than in Victoria and with greater awareness of the generally mild nature of pH1N1 disease, found greater frequency of excursions outside the home (75%) than did our survey [20]. Unlike our sample, the WA study included 'peers' as well as those children identified as actual 'contacts'. The latter were more likely to stay at home than their unexposed friends, exceeded only by cases, of whom there were relatively few [20]. Frequent socialization was reported among students sent home during pH1N1 driven closures in the United States (US) [18], in keeping with earlier observations during a large seasonal influenza B epidemic, in which individual risk perception was assessed and reported to be low [17]. Australian surveys have found a lower anticipated compliance with voluntary quarantine measures for seasonal influenza infection, compared with a pandemic virus [21].

Parental care in the home was associated with higher compliance with social restrictions. During pH1N1 associated elementary school closures in Pennsylvania, only one in five parents took time off work to care for children despite dual income earners in two thirds of households. In that study, 69% of affected children made excursions to locations outside the home during the closure period [22]. A recent contact diary study reported a 50% reduction in child socialization during school holiday periods in the United Kingdom (UK) compared with term time, suggested to be predictive of behavior during a public health intervention [23]. However, the relevance of this finding to an emergency school closure setting should be interpreted with caution, as making 'ad hoc' arrangements for child care at short notice may lead to very different patterns of child socialization, compared with periods of scheduled leave.

Oseltamivir was well accepted by respondents in this study, with almost all taking at least half of the course, and very few reporting side effects. In a 'real-time' survey from the UK, just under half of secondary school students and three quarters of primary school students completed a prescribed course of oseltamivir [19]. Non-compliance was ascribed to gastro-intestinal side-effects in half, and may have been more reliably reported than in our study due to an absence of recall bias, although questionnaires were only completed by around 40% of the sample population [19]. Similarly high rates of adverse events were seen among children receiving oseltamivir in a comprehensive school in the South-West of England, but with better compliance and a higher study participation rate (> 90%) [24].

## Conclusions

High levels of compliance with quarantine and antiviral recommendations were observed in our study population, derived from families affected by school closures in Victoria during the early days of the 2009 H1N1 epidemic. These estimates likely reflect a 'best case' scenario, fuelled by high levels of public awareness and anxiety at the time the measures were imposed. However, the complex nature of the intervention was reflected in the variable directives received by families, which likely undermined its impact.

In related work, we explore the predictors of compliance at household level in further detail, including socio-economic status and parental employment arrangements, along with financial consequences of home quarantine recommendations for the family (Prof Anne Kavanagh, personal communication). At societal level, the costs associated with school closures are substantial [7], making their economic justification difficult in the absence of high case fatality, even where highly effective [13]. As implemented, the measures in Victoria were unlikely to have substantially altered the course of the epidemic. This study emphasizes the need to understand the feasibility of public health measures when considering their likely health and economic impacts in real world settings.

## List of Abbreviations Used

AHMPPI: Australian Health Management Plan for Pandemic Influenza; CI: Confidence interval; pH1N1: Pandemic influenza A (H1N1) 2009; UK: United Kingdom; US: United States of America; WA: Western Australia

## Competing interests

The authors declare that they have no competing interests.

## Authors' contributions

All authors were involved in study and questionnaire design. SP and PN liased with schools that distributed study information to parents. KM and JMcV analysed the study data. JMcV led drafting of the manuscript. All authors read and approved the final manuscript.

## Pre-publication history

The pre-publication history for this paper can be accessed here:

http://www.biomedcentral.com/1471-2334/11/257/prepub
